# Supplementary material for: Regional β-Diversity of Stream Insects in Coastal Alabama Is Correlated with Stream Conditions, Not Distance among Sites
Source: Insects. 2023 Oct 31;14(11):847. doi: 10.3390/insects14110847 (PMC10671468; doi:10.3390/insects14110847)
Supplement: Supplementary file 1 [file insects-14-00847-s001.zip › insects-2671965-supplementary.pdf]

Table S1. Mean stream conditions for stream insect collections, Mobile and Baldwin counties, Alabama for both summer and fall collections.

	Variables	Mean (±SE) [1]	Range
**Summer 2007**	Depth (m)	0.31 ± 0.044	0.05-0.54
	Velocity (m/s)	0.03 ± 0.003	0.02-0.07
	Width (m)	4.3 ± 0.40	2.0-8.0
	Discharge (m^3/s)	0.05 ± 0.008	0.01-0.12
	Conductivity ($\mu S\ cm^{-1}$, 25 °C)	35.7 ± 4.04	18.8-78.6
	Hardness (ppm)	8.23 ± 0.763	4.9-14.0
	Temperature (°C)	23.3 ± 0.82	13.6-29.3
	pH	6.05 ± 0.105	5.2-7.0
	Elevation (m)	15.0 ± 2.09	3.0-34.0
	% Dissolved Oxygen	91.1 ± 1.83	77.5-100.0
	Canopy Cover	2.0	1.5-3.0
	Riparian Vegetation	3.0	2.0-3.0
	Streambed-particle Size	2.0	1.5-2.0
**Fall 2007**	Depth (m)	0.30 ± 0.023	0.11-0.54
	Velocity (m/s)	0.31 ± 0.021	0.13-0.57
	Width (m)	4.3 ± 0.37	2.0-9.0
	Discharge (m^3/s)	0.41 ± 0.057	0.10-1.10
	Conductivity ($\mu S\ cm^{-1}$, 25 °C)	34.9 ± 2.45	18.4-61.9
	Hardness (ppm)	8.4 ± 1.14	3.2-25.0
	Temperature (°C)	18.1 ± 0.73	10.9-24.4
	pH	6.0 ± 0.10	4.7-7.0
	Elevation (m)	16 ± 1.94	3.0-34.0
	% Dissolved Oxygen	90.2 ± 1.30	75.6-100.0
	Canopy Cover	2.0	2.0-3.0
	Riparian Vegetation	3.0	2.0-3.0
	Streambed-particle size	2.0	1.5-2.5

[1] Canopy cover (1-3) and Riparian Vegetation (1-3) and Streambed-particle size (1-4) are ranked variables following McCreadie et al. [31]. Therefore medians, not means, are given.

Table S2. All genera found in summer (29 May to 29 June, 2007) and fall (2 October to 17 November, 2007) collections.

Fall		Summer	
Genera	% occurrence among sites	Genera	% occurrence among sites
Ancyronyx	94.1176	Ancyronyx	100.000
Boyeria	94.1176	Stenelmis	95.652
Stenelmis	94.1176	Argia	91.304
Dubiraphia	82.3529	Chimarra	91.304
Leuctra	82.3529	Hydropsyche	91.304
Gomphus	76.4706	Progomphus	91.304
Chimarra	70.5882	Cheumatopsyche	86.957
Gonielmis	70.5882	Acroneuria	82.609
Acroneuria	64.7059	Leuctra	82.609
Neoperla	64.7059	Gomphus	78.261
Neurocordulia	64.7059	Neurocordulia	78.261
Cheumatopsyche	58.8235	Oecetis	78.261
Macromia	58.8235	Anisocentropus	73.913
Progomphus	58.8235	Gonielmis	73.913
Perlesta	52.9412	Microcylloepus	73.913
Brachycentrus	47.0588	Dubiraphia	69.565
Dineutus	47.0588	Perlinella	69.565

Hydropsyche	47.0588	Boyeria	65.217
Microcylloepus	47.0588	Brachycentrus	65.217
Anisocentropus	41.1765	Paragnetina	65.217
Argia	41.1765	Ectopria	56.522
Cordyalus	35.2941	Calopteryx	52.174
Hydroptila	35.2941	Neuroclipsis	52.174
Nigronia	35.2941	Lype	47.826
Nectopsyche	29.4118	Macromia	47.826
Oecetis	29.4118	Nectopsyche	47.826
Paragnetina	29.4118	Nyctiophylax	47.826
Sialis	29.4118	Corydalus	43.478
Dromogomphus	23.5294	Macrostemum	43.478
Enallagma	23.5294	Agarodes	34.783
Lioporeus	23.5294	Dineutus	34.783
Hagenius	17.6471	Dromogomphus	30.435
Helocordula	17.6471	Nigronia	30.435
Hydrochus	17.6471	Sialis	30.435
Hygrotus	17.6471	Taeniopteryx	30.435
Macronychus	17.6471	Triaenodes	30.435
Molanna	17.6471	Enallagma	26.087
Perlinella	17.6471	Cernotina	21.739
Berosus	11.7647	Diplectrona	21.739
Calopteryx	11.7647	Hydroptila	21.739
Cordulagaster	11.7647	Macronychus	21.739
Diplectrona	11.7647	Phylocentropus	21.739
Ectopria	11.7647	Berosus	17.391
Heteroplectron	11.7647	Lioporeus	17.391

Lype	11.7647	Ceraclea	13.043
Macrostenum	11.7647	Gyrinus	13.043
Micrasema	11.7647	Lepidostoma	13.043
Neoporus	11.7647	Molanna	13.043
Oxyethira	11.7647	Neoporus	13.043
Sperchopsis	11.7647	Polycentropus	13.043
Bidessonotus	5.8824	Helocordulia	8.696
Cernotina	5.8824	Heteroplectron	8.696
Cymbiodyta	5.8824	Mayatrichia	8.696
Gyrinus	5.8824	Neoperla	8.696
Helocombus	5.8824	Neotrichia	8.696
Mayatrichia	5.8824	Perlesta	8.696
Neureclipsis	5.8824	Sperchopsis	8.696
Nyctiophylax	5.8824	Climacia	4.348
Phylocentropus	5.8824	Cordulagaster	4.348
Pycnopsyche	5.8824	Hagenius	4.348
Sisyra	5.8824	Hydrochus	4.348
		Micrasema	4.348
		Oxyethira	4.348
		Pteronarcys	4.348
		Ptilostomis	4.348
		Rhyacophila	4.348
No. of genera = 61		No. of genera = 66	
No. of sites = 17		No. of sites = 23	
